# Access to substance use treatment among young adults who use prescription opioids non-medically

**DOI:** 10.1186/s13011-016-0082-1

**Published:** 2016-11-29

**Authors:** Elliott J. Liebling, Jesse L. Yedinak, Traci C. Green, Scott E. Hadland, Melissa A. Clark, Brandon D. L. Marshall

**Affiliations:** 1 Department of Epidemiology, Brown University School of Public Health, 121 South Main Street, Box G-S-121-4, Providence, RI 02912 USA; 2 Department of Emergency Medicine, Boston University School of Medicine, 771 Albany Street, Room 1208, Boston, MA 02118 USA; 3 The Warren Alpert School of Medicine of Brown University, Rhode Island Hospital, 55 Claverick Street, Providence, RI 02903 USA; 4 Division of General Pediatrics, Department of Pediatrics, Boston University School of Medicine, 88 East Newton Street, Vose Hall Room 322, Boston, MA 02118 USA; 5 Division of Adolescent/Young Adult Medicine, Department of Medicine, Boston Children’s Hospital, 333 Longwood Avenue, Boston, MA 02115 USA; 6 Department of Pediatrics, Harvard Medical School, 25 Shattuck Street, Boston, MA 02115 USA; 7 Department of Quantitative Health Sciences & Center for Health Policy and Research, University of Massachusetts Medical School, 55 Lake Ave North, Worcester, MA 01605 USA

**Keywords:** Substance use, Treatment, Young adults, Prescription opioids, Non-medical use, Access, Utilization, Barriers

## Abstract

**Background:**

Non-medical prescription opioid (NMPO) use is a substantial public health problem in the United States, with 1.5 million new initiates annually. Only 746,000 people received treatment for NMPO use in 2013, demonstrating substantial disparities in access to treatment. This study aimed to assess correlates of accessing substance use treatment among young adult NMPO users in Rhode Island, a state heavily impacted by NMPO use and opioid overdose.

**Methods:**

This analysis uses data from a study of 200 Rhode Island residents aged 18 to 29 who reported NMPO use in the past 30 days. We compared individuals who had ever successfully enrolled in a substance use treatment program without ever facing barriers, individuals who had ever attempted to enroll but were unable, and individuals who never attempted to enroll. We used multinomial logistic regression to determine the independent correlates of never attempting and unsuccessfully attempting to access substance use treatment.

**Results:**

Among 200 participants, the mean age was 24.5, 65.5% were male, and 61.5% were white. Nearly half (45.5%) had never attempted to enroll in substance use treatment, while 35.0% had successfully enrolled without ever facing barriers and 19.5% were unsuccessful in at least one attempt to enroll. In multivariable models, non-white participants were more likely to never have attempted to enroll compared to white participants. Previous incarceration, experiencing drug-related discrimination by the medical community, and a monthly income of $501 - $1500 were associated with a decreased likelihood of never attempting to enroll. A history of overdose and a monthly income of $501 - $1500 were associated with an increased likelihood of unsuccessfully accessing treatment. The most commonly reported barriers to accessing treatment were waiting lists (*n* = 23), health insurance not approving enrollment (*n* = 20), and inability to pay (*n* = 16).

**Conclusions:**

This study demonstrates significant disparities in access to treatment among young adults who report NMPO use. A history of overdose was shown to correlate with experiencing barriers to substance use treatment utilization. Interventions are needed to reduce drug-related discrimination in clinical settings and to provide mechanisms that link young adults (particularly with a history of overdose) to evidence-based treatment.

## Background

Non-medical prescription opioid (NMPO) use—defined as intentional use of one’s own opioid prescription outside of prescribed parameters or use of an opioid without a prescription [1, 2]—has become a major public health problem in the United States [3]. There were 4.3 million active NMPO users in the United States in 2014 (representing 1.6% of the population), and 1.5 million new initiates in 2013 [4, 5]. Opioid overdose has become an epidemic in the United States, overtaking motor vehicle crash mortality in 2009 [6]. There were 18,893 opioid overdose deaths in 2014 compared to 16,235 in 2013, a year-over-year increase of 16% [7]. Yearly, opioid overdose costs $20.4 billion due to lost productivity and medical expenses [8]. Rhode Island has been heavily impacted by NMPO use and opioid overdose. In 2015, 258 individuals died of an overdose, more than deaths due to homicide, motor vehicle accidents, and suicide combined [9, 10].

The prevalence of NMPO use is particularly high among young adults; the percentage of users was highest among 18- to 25-year-olds, reaching 2.8% in 2014 [4]. These rates of NMPO use trail only marijuana as the most commonly reported drug of use among young adults [4]. Among young adults, NMPO use has been associated with transitioning to heroin use [11, 12]. Although many individuals may initially receive opioids through a prescription, adults ages 18 and above who first obtain opioids illegally are more likely to transition to heroin use [13–15].

A number of interventions, including state legislation and clinical guidelines, have been shown to decrease the public health burden associated with NMPO use [16, 17]. For example, opioid use disorders can be treated effectively with medication-assisted treatment (MAT) with methadone, buprenorphine, or naltrexone [18, 19]. However, many of these strategies are grossly under-utilized across the United States. Only 746,000 people in the United States received treatment for NMPO use in 2013, representing a sizable gap between utilization of care and total reported use [5]. Nonetheless, this was more than double the number of people who received care in 2002 [5]. Among youth with substance use disorders, only one in ten individuals in the United States receives treatment [20].

**Fig. 1:**
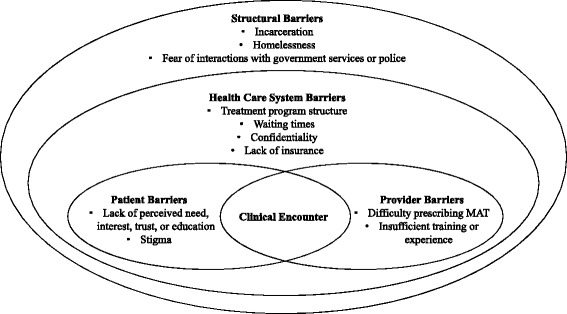
A multilevel approach to potential barriers to accessing substance use treatment among young NMPO users. NMPO: Non-medical prescription opioid use. MAT: Medication-assisted treatment. Adapted from [21]

The conceptual framework used to guide this paper is adapted from the multilevel factors approach presented by Kilbourne et al. [21]. This health services-oriented approach to health disparities identifies three primary determinants of health care access disparities—patient factors, provider factors, and health care system factors, as well as clinical encounters between patients and providers (see Fig. 1). Given that this conceptual framework focuses on factors within the health care system, we have adapted the model to additionally consider barriers from outside of the system, such as interactions with government services or police.

### Patient-level factors and barriers

Research surrounding substance use treatment-seeking behavior among adults is well established [22–24]. However, fewer studies have examined the treatment experiences of young people who use prescription opioids non-medically. Young adults ages 18 to 25 who report NMPO use and perceive the need for treatment report barriers to utilizing treatment, including an unwillingness or lack of readiness to stop using, fear of negative opinions when others find out about substance use (e.g., parents), a perceived ability to handle the problem on their own, and a lack of knowledge regarding where to obtain youth-friendly services [25]. Young NMPO users have also shown lower rates of health education and trust in health care providers compared to adult NMPO-using populations [26, 27].

### Provider-level factors and barriers

People who use opioids non-medically may not seek substance use treatment due to important provider-level factors such as perceived stigma, discrimination, and other barriers experienced during clinical encounters [28]. Adult NMPO users often have difficulty accessing MAT in particular due to prescribing practices [29–31]. Prescribing buprenorphine requires physicians to obtain a federal waiver, complete an eight-hour training, and meet other criteria [30]. Prescribing buprenorphine is limited by difficulties with reimbursement and professional training, leading to a lack of prescribing physicians in many settings throughout the United States [29, 31–33]. Provision of methadone is similarly restricted by federal policies that limit access for many patients [34]. For young adults, treatment may not include MAT due to the fact that many pediatricians do not have experience managing patients on these medications or are not approved to prescribe specific opioid agonist therapies (i.e., buprenorphine) [20, 33].

### Health care system factors and barriers

Barriers to substance use treatment operating at the health care system-level are present for all age groups. Factors and barriers at the health care system-level include treatment program structure, waiting times, and concerns regarding confidentiality [21, 35–38].

Individuals who are considering entering substance use treatment may be hesitant due to doubt surrounding their ability to meet a program’s criteria, including supervision, attendance, and abstinence [37]. Furthermore, waiting lists represent a critical barrier to accessing treatment [39, 40]. There is often a wide divergence between expected wait time among NMPO users and the actual wait times that exist. A survey of 85 daily or almost daily opioid users conducted in New Zealand showed that participants eligible for opioid substitution therapy estimated waiting times on average to be 6.5 days, whereas the actual mean waiting time was 4.4 months [38]. Total waiting times for substance use treatment in the United States can also reach over two months [40]. In young adult NMPO-using populations, waiting times, particularly for methadone clinics, remain understudied [29].

Young adults with substance use disorders face numerous health care system barriers, including lack of insurance, insufficiently integrated delivery systems, underfunding of programs, and issues with patient confidentiality [36]. Furthermore, young adults may also fear that providers will not or cannot keep their substance use confidential from parents or other caregivers, reducing their likelihood of seeking care [36].

### Structural barriers

There are additional barriers to accessing substance use treatment that exist outside of the health care system. In some settings, NMPO users may choose not to seek treatment due to a fear of disclosing illegal behavior to the police, government, or other organizations [41]. Incarceration, a structural factor, can act as a barrier by interrupting treatment access among young adults who use opioids non-medically [42]. However, incarceration may also facilitate access to substance use treatment through referrals and linkages to community-based providers [43]. Finally, previous studies have shown that young adults who are homeless also have less success accessing social services [44].

### Objectives and hypothesis

This study aimed to assess patient-level, provider-level, health care system, and structural factors associated with substance use treatment access among young adults who use prescription opioids non-medically. We also sought to determine the risk factors associated with ever experiencing barriers while attempting to access substance use treatment services. Our hypothesis was that patient-specific, provider-centered, and health care system barriers prevent young adults from successfully enrolling in care.

## Methods

### Study design and sample selection

This analysis uses data from the Rhode Island Young Adult Prescription Drug Study (RAPiDS), a pilot study of 200 Rhode Island residents aged 18 to 29 who reported use of non-medical use of prescription opioids in the past 30 days. Participants were recruited between January 2015 and February 2016 through a combination of targeted canvassing (TARC) and mixed internet-based recruitment (MIBR). Specifically, recruitment for RAPiDS was divided between two phases. In the first phase, eligible participants were recruited via TARC. This recruitment method relied primarily on bus advertisements and flyering in areas where drug-using young adults were known to congregate. During this phase, individuals who completed the survey were also encouraged to refer friends and acquaintances to the study. The second phase utilized MIBR as a recruitment method, which included regular posts on online classified sites (e.g., Craigslist), social media (i.e., Facebook), and forums (e.g., Reddit).

In order to be eligible to participate in the study, individuals needed to meet five criteria. These were: being aged 18 to 29 at the time of the interview, residing in Rhode Island, not being currently involved in formal alcohol or substance use treatment, being able to complete an interview in English, and providing informed consent. Additionally, participants were required to confirm non-medical use of prescription opioids in the past 30 days by indicating which opioids he or she recently used non-medically, based on a modified version of the Substance Abuse and Mental Health Service Administration’s “pill card A” [45]. No further eligibility restrictions were specific to this analysis.

Eligible participants completed a computer-assisted interview of approximately 45 min with a trained interviewer. Sensitive portions of the survey were self-administered using a computer. Individuals were offered $25 for participating in the study. The Brown University Institutional Review Board approved the study protocol.

### Assessment of treatment utilization

The primary outcome for this analysis was self-reported history of substance use treatment utilization. To define this outcome, we divided the sample into three mutually exclusive and collectively exhaustive groups. These groups were conceptually defined as: (1) individuals who had never attempted to enroll in substance use treatment in their lifetime, (2) individuals who reported ever enrolling in substance use treatment without ever being unsuccessful in attempting to enroll, and (3) individuals who were unsuccessful in at least one attempt to access substance use treatment, regardless of whether or not they had ever been in treatment. The operational definition of this outcome was a combination of two self-reported binary variables. The first question asked if the participant had ever been in any kind of alcohol or drug treatment program (yes vs. no), which was defined as detox, medication-assisted therapy, a group program (i.e., 12-step AA or NA programs), outpatient day treatment, or a residential drug treatment program. A follow-up question asked all participants whether she/he had ever tried to enroll in a substance use treatment program but were unable to (yes vs. no).

### Independent variables of interest

The RAPiDS survey instrument measured sociodemographic characteristics, patient-level factors, provider-level factors, health care system factors, and structural factors. The selection of variables to include in the analysis was guided by our conceptual model and the a priori hypotheses described above. Specifically, the analysis included measures of sociodemographic factors such as age, sex, ethnicity, race, sexual orientation, educational attainment, employment, personal income, and geographic residence. Age was defined as a continuous variable measured by year, ranging from 18 to 29. Sex was defined as a binary measure of sex at birth (male vs. female). Ethnicity was defined as being of Hispanic or Latino descent (yes vs. no). The race variable included the possible responses: American Indian or Alaska Native, Asian, black (African, Haitian, or of Cape Verdean descent), Native Hawaiian or other Pacific Islander, white, mixed, bi-racial, or multi-racial, and something else. For the purpose of these analyses, the categories were collapsed into white and non-white races. Sexual orientation was also collapsed into two categories of straight vs. gay, lesbian, bisexual, queer, or something else. Educational attainment was measured by highest level of education received. Employment was defined as being currently employed full-time or part-time (yes vs. no). Personal income was reported as monthly gross take home income, including from public assistance or family support. Geographic residence type used self-reported current ZIP code or town of residence and was categorized as urban, suburban, or rural according to standard US census definitions and the Rhode Island land use survey [46].

We included any prior diagnoses of mental illness as additional patient-level factors. Specifically, participants were asked if she/he had ever been diagnosed with: attention deficit hyperactivity disorder or attention deficit disorder, obsessive-compulsive disorder, an eating disorder, a depressive disorder, bipolar disorder, an anxiety disorder, psychosis, or another diagnosis. Additional patient-level factors included in the analysis were ever having overdosed by accident, ever having used heroin, ever having used cocaine, and ever having used a needle to chip, fix, muscle, or inject drugs.

The provider-level factors included in the analysis were past history of being prescribed an opioid and ever experiencing drug-related discrimination by the medical community. Specifically, participants were asked to report if she/he had felt discriminated against by the medical community, such as a doctor, nurse, or clinic staff, due to their drug use. This measure was adapted from an item validated in a previous study examining stigma, discrimination, and devaluation experienced by people who use drugs [47].

We included self-reported current health insurance status (yes vs. no) as a health care system-level factor. Homelessness, a structural factor, was defined as ever having been homeless. Other structural factors, such as history of juvenile detention, arrest, and incarceration, were assessed by asking individuals if she/he had ever been detained in a juvenile detention center or training school, ever been arrested, ever been detained or incarcerated in an adult jail, and ever been incarcerated in a prison. Finally, participants were asked to report their main barriers to accessing addiction treatment. The list of possible response options was generated based on a review of past literature that has examined barriers to substance use treatment among young adults, and included primarily health care system barriers, such as waiting lists, issues with health insurance, and programs that were not youth-friendly or otherwise turned individuals down [36, 40]. Participants could also state barriers that were not listed.

### Statistical analyses

First, we used descriptive statistics to summarize the characteristics of the complete sample and each of the three subsamples. Next, we conducted Pearson’s chi-squared tests for categorical variables and ANOVA for continuous variables in order to assess differences for each variable across the three samples. We used Fisher’s exact test when one or more of the cells included fewer than 5 observations.

Third, we constructed a multivariable model using multinomial logistic regression. In the model, individuals who have never attempted to enroll (*n* = 91) and individuals who were unsuccessful in at least one attempt to access substance use treatment (*n* = 39) were compared against individuals who have ever successfully enrolled in substance use treatment without ever being unable to enroll (*n* = 70). Variables that were found to be significant at the *p* < 0.05 level in Table 2 were included in the original model, as well as sex and recruitment source. To obtain a more parsimonious model, we first calculated the variance inflation factor for all sets of variables and removed those that were found to be collinear. Next, a backwards elimination stepwise process was applied until at least one of the effect estimates for each variable was significant at *p* < 0.05. The final model was also adjusted for recruitment source (TARC vs. MIBR), and all *p*-values are two-sided. Analyses were conducted using Stata SE 13.1.

## Results

### Descriptive statistics

Among 200 participants, the mean age was 24.5 (SD = 3.24) and 65.5% (*n* = 131) of the sample was male. In the sample, 14.0% (*n* = 28) of the participants were of Hispanic or Latino descent. The majority (*n* = 123, 61.5%) was white and 38.5% (*n* = 77) were of another race. Among the participants, 86.0% (*n* = 172) of the sample reported sexual orientation as straight, and 13.5% were lesbian, gay, bisexual, or queer (LGBQ) and other. Among participants, 11.5% (*n* = 23) had less than a high school education and 55.7% (*n* = 108) were unemployed.

The distribution of other patient, provider, health-systems, and structural variables of interest are provided in Table 1. Of note, the majority (*n* = 175, 87.5%) of the sample had health insurance and had ever been homeless (*n* = 109, 54.5%). Among participants, 29.5% (*n* = 59) reported having experienced drug-related discrimination by the medical community.

**Table 1:** Characteristics of RAPiDS participants (*n* = 200)

	Participants 200 (100%) n (%)
Mean Age (Standard deviation)	24.5 (3.24)
Sex	
Male	131 (65.5%)
Female	69 (34.5%)
Ethnicity	
Hispanic or Latino descent	28 (14.0%)
Non-Hispanic	172 (86.0%)
Race	
White	123 (61.5%)
Non-white	77 (38.5%)
Sexual orientation	
Straight	172 (86.0%)
LGBQ and other	27 (13.5%)
Education	
Less than high school	23 (11.5%)
High school/GED	76 (38.0%)
Beyond high school	101 (50.5%)
Employment status	
Full or part-time	88 (44.0%)
Unemployed	108 (55.7%)
Monthly income	
< $501	104 (52.0%)
$501 - $1500	58 (29.0%)
> $1500	35 (17.5%)
Geographic residence type	
Rural	23 (11.5%)
Suburban	19 (9.5%)
Urban	151 (75.5%)
Ever overdosed by accident	
Yes	53 (26.5%)
No	147 (73.5%)
Ever used heroin	
Yes	85 (42.5%)
No	115 (57.5%)
Ever used cocaine	
Yes	133 (66.5%)
No	67 (33.5%)
Ever used a needle to inject drugs	
Yes	59 (29.5%)
No	140 (70.0%)
Ever diagnosed with…	
ADHD/ADD	78 (39.0%)
Depression	95 (47.5%)
Anxiety	98 (49.0%)
Depression and anxiety	73 (37.0%)
Other (OCD, eating disorders, bipolar, etc.)	58 (29.0%)
Ever prescribed opioid	
Yes	121 (61.1%)
No	77 (38.9%)
Drug-related discrimination by medical community	
Yes	59 (29.5%)
No	141 (70.5%)
Insurance status	
Yes	175 (87.5%)
No	25 (12.5%)
Ever detained in juvenile detention center	
Yes	48 (24.0%)
No	152 (76.0%)
Ever arrested	
Yes	141 (70.5%)
No	59 (29.5%)
Ever incarcerated in jail or prison	
Yes	94 (47.0%)
No	106 (53.0%)
Ever homeless	
Yes	109 (54.5%)
No	91 (45.5%)
Recruitment method	
Field	79 (39.5%)
Internet	119 (59.5%)

*Notes*: Not all columns sum to 100% due to missing data and/or rounding

*LGBQ* Lesbian, gay, bisexual, or queer. *GED* General Education Development

*ADHD/ADD* Attention deficit hyperactivity disorder/attention deficit disorder

*OCD* Obsessive-compulsive disorder

Approximately half (*n* = 91, 45.5%) of the sample had never attempted to enroll in substance use treatment, while 35.0% (*n* = 70) had ever successfully enrolled without being unable to and 19.5% (*n* = 39) were unsuccessful in at least one attempt to access treatment. Therefore, among individuals who had ever attempted to access substance use treatment (*n* = 109), 35.8% (*n* = 39) experienced barriers preventing enrollment at least once. Of the 39 individuals who were unsuccessful in at least one attempt to access treatment, 36 (92.3%) were successful in another attempt to enroll. Among participants who reported ever having successfully enrolled in substance use treatment, the reported types of substance use treatment received were an outpatient drug or alcohol treatment program (*n* = 67, 63.2%), a self-help group (*n* = 61, 57.6%), detox (*n* = 57, 53.8%), a residential drug treatment program (*n* = 51, 48.1%), methadone or buprenorphine/naloxone treatment (*n* = 48, 45.3%), a day treatment program or partial hospitalization program (*n* = 25, 23.6%), and a transitional halfway house (*n* = 1, 0.9%).

Among participants who were unsuccessful in enrolling (*n* = 39), the most commonly reported barriers to accessing substance use treatment were a waiting list (*n* = 23, 59.0%), health insurance not allowing enrollment (*n* = 20, 51.3%), an inability to pay (*n* = 16, 41.0%), being turned down by a program (*n* = 12, 30.8%), not having health insurance (*n* = 8, 20.5%), no treatment programs being nearby (*n* = 8, 20.5%), not knowing of any programs (*n* = 3, 7.7%), and programs not being youth-friendly (*n* = 3, 7.7%). Participants stated four barriers outside of the provided list; each had one response.

### Correlates of accessing substance use treatment

Comparisons of the three treatment access groups are shown in Table 2. We observed significant racial differences across the three groups. For example, only 27.1% of those who had successfully enrolled in treatment without ever facing barriers were non-white, compared to 54.9% of those who had never attempted to access treatment and 20.5% of those who had ever unsuccessfully attempted to enroll in treatment. Additionally, over half (56.4%) of those who had ever been unsuccessful in an attempt to enroll in treatment reported ever experiencing drug related discrimination by the medical community, compared to only 37.1% of those who had successfully enrolled in treatment.

**Table 2:** Description of RAPiDS participants by history of utilization of substance use treatment (*n* = 200)

		Attempted to enroll (*n* = 109)			
	Never attempted to enroll 91 (45.5%) n (%)	Successfully enrolled without barriers 70 (35.0%) n (%)	Unsuccessfully attempted to enroll 39 (19.5%) n (%)	*χ* ^2^ (df)	*p* - value
Mean Age (Standard deviation)	23.7 (3.23)	24.9 (3.30)	25.8 (2.60)	6.97 (2)^a^	0.001
Sex				5.44 (2)	0.066
Male	52 (57.1%)	52 (74.3%)	27 (69.2%)		
Female	39 (42.9%)	18 (25.7%)	12 (30.8%)		
Ethnicity				(2)^b^	0.044
Hispanic or Latino descent	14 (15.4%)	13 (18.6%)	1 (2.6%)		
Non-Hispanic	77 (84.6%)	57 (81.4%)	38 (97.4%)		
Race				19.54 (2)	<0.001
White	41 (45.1%)	51 (72.9%)	31 (79.5%)		
Non-white	50 (54.9%)	19 (27.1%)	8 (20.5%)		
Sexual orientation				(2)^b^	0.431
Straight	75 (82.4%)	61 (87.1%)	36 (92.3%)		
LGBQ and other	15 (16.5%)	9 (12.9%)	3 (7.7%)		
Education				2.46 (4)	0.652
Less than high school	11 (12.1%)	6 (8.6%)	6 (15.4%)		
High school/GED	32 (35.2%)	31 (44.3%)	13 (33.3%)		
Beyond high school	48 (52.7%)	33 (47.1%)	20 (51.3%)		
Employment Status				4.34 (2)	0.114
Full or part-time	46 (50.6%)	24 (34.3%)	18 (46.2%)		
Unemployed	45 (49.5%)	46 (65.7%)	21 (53.9%)		
Monthly income				15.84 (4)	0.003
< $501	39 (43.3%)	48 (70.6%)	17 (43.6%)		
$501 - $1500	35 (38.5%)	9 (12.9%)	14 (35.9%)		
> $1500	16 (17.6%)	11 (15.7%)	8 (20.5%)		
Geographic residence type				5.62 (4)	0.229
Rural	9 (10.1%)	6 (9.2%)	8 (20.5%)		
Suburban	6 (6.7%)	8 (12.3%)	5 (12.8%)		
Urban	74 (83.1%	51 (78.5%)	26 (66.7%)		
Ever overdosed by accident				24.99 (2)	<0.001
Yes	10 (11.0%)	23 (32.9%)	20 (51.3%)		
No	81 (89.0%)	47 (67.1%)	19 (48.7%)		
Ever used heroin				58.87 (2)	<0.001
Yes	13 (14.3%)	41 (58.6%)	31 (79.5%)		
No	78 (85.7%)	29 (41.4%)	8 (20.5%)		
Ever used cocaine				(2)^b^	<0.001
Yes	37 (40.7%)	61 (87.1%)	35 (89.7%)		
No	54 (59.3%)	9 (12.9%)	4 (10.3%)		
Ever used a needle to inject drugs				(2)^b^	<0.001
Yes	4 (4.4%)	28 (40.0%)	27 (69.2%)		
No	86 (94.5%)	42 (60.0%)	12 (30.8%)		
Ever diagnosed					
ADHD/ADD	27 (29.7%)	31 (44.3%)	20 (51.3%)	6.62 (2)	0.036
Depression	29 (31.9%)	43 (61.4%)	23 (59.0%)	16.42 (2)	<0.001
Anxiety	37 (40.7%)	36 (51.4%)	25 (64.1%)	6.26 (2)	0.044
Other	22 (24.2%)	21 (30.0%)	15 (38.5%)	2.76 (2)	0.252
Ever prescribed opioid				0.24 (2)	0.886
Yes	53 (59.6%)	43 (61.4%)	25 (64.1%)		
No	36 (40.5%)	27 (38.6%)	14 (35.9%)		
Drug-related discrimination by medical community				28.8 (2)	<0.001
Yes	11 (12.1%)	26 (37.1%)	22 (56.4%)		
No	80 (87.9%)	44 (62.9%)	17 (43.6%)		
Insurance status				(2)^b^	0.260
Yes	79 (86.8%)	59 (84.3%)	37 (94.9%)		
No	12 (13.2%)	11 (15.7%)	2 (5.1%)		
Ever detained in juvenile detention center				11.29 (2)	0.004
Yes	13 (14.3%)	19 (27.1%)	16 (41.0%)		
No	78 (85.7%)	51 (72.9%)	23 (59.0%)		
Ever arrested				16.90 (2)	<0.001
Yes	51 (56.0%)	57 (81.4%)	33 (84.6%)		
No	40 (44.0%)	13 (18.6%)	6 (15.4%)		
Ever incarcerated in jail or prison				17.68 (2)	<0.001
Yes	28 (30.8%)	42 (60.0%)	24 (61.5%)		
No	63 (69.2%)	28 (40.0%)	15 (38.5%)		
Ever homeless				12.03 (2)	0.002
Yes	40 (44.0%)	39 (55.7%)	30 (76.9%)		
No	51 (56.0%)	31 (44.3%)	9 (23.1%)		
Recruitment method				13.11 (2)	0.001
Field	24 (27.0%)	32 (45.7%)	23 (59.0%)		
Internet	65 (73.0%)	38 (54.3%)	16 (41.0%)		

Significance ascertained using a chi-square test unless otherwise noted

^a^Significance tested using an ANOVA

^b^Significance ascertained using a Fisher’s exact test

*Notes*: Not all columns sum to 100% due to missing data and/or rounding

*LGBQ* Lesbian, gay, bisexual, or queer

*GED* General Education Development

*ADD/ADHD* Attention deficit disorder/attention deficit hyperactivity disorder

The variance inflation factor between race and ever using heroin was 1.32, demonstrating moderate collinearity. Additionally, 61.8% of white participants ever used heroin, compared to only 10.4% of non-white participants. The variance inflation factor between ever using heroin and ever using a needle to inject drugs was 1.88, demonstrating moderate collinearity. Ever using cocaine was also moderately correlated with race and ever using heroin, with variance inflation factors of 1.27 and 1.53, respectively. Finally, the variance inflation factor between diagnoses of an anxiety disorder and a depressive disorder was 1.43, demonstrating moderate collinearity. As such, heroin use, cocaine use, lifetime injection drug use, and ever being diagnosed with a depressive disorder were excluded from the final multivariable model. The variance inflation factor between past incarceration and detention in a juvenile detention center was 1.15, demonstrating low collinearity.

In the final model, factors independently associated with never enrolling in substance use treatment compared to participants who had successfully accessed treatment without ever facing barriers are shown in Table 3. Compared to white participants, non-white participants were significantly more likely to have never attempted to enroll in substance use treatment, with over three times the adjusted risk of never attempting to enroll compared to successfully enrolling without ever facing barriers. Compared to a monthly income of less than $501, a monthly income of $501 - $1500 was associated with almost four times the adjusted risk of never attempting to enroll in substance use treatment compared to successfully enrolling in substance use treatment without ever facing barriers. This was among the strongest associations found. Being of Hispanic or Latino descent, ever experiencing drug-related discrimination by the medical community, and previous incarceration were associated with a decreased risk of never enrolling in substance use treatment compared to successfully enrolling in substance use treatment without ever facing barriers, adjusted for the other covariates.

**Table 3:** Adjusted risk ratios of substance use treatment enrollment outcomes vs. successfully enrolling: RAPiDS (*n* = 200)

	Never attempted to enroll (*n* = 91)			Unsuccessfully attempted to enroll (*n* = 39)		
	Adjusted risk ratio	95% Confidence Interval (CI)	*p* - value	Adjusted risk ratio	95% Confidence Interval (CI)	*p* - value
Ethnicity						
Hispanic or Latino descent	0.30	(0.10, 0.95)	0.040	0.12	(0.01, 1.07)	0.058
Non-Hispanic	1.00	(Ref)		1.00	(Ref)	
Race						
White	1.00	(Ref)		1.00	(Ref)	
Non-white	3.16	(1.28, 7.83)	0.013	1.39	(0.44, 4.43)	0.578
Monthly income						
< $501	1.00	(Ref)		1.00	(Ref)	
$501 - $1500	3.93	(1.53, 10.12)	0.005	5.36	(1.79, 16.03)	0.003
> $1500	2.16	(0.90, 5.80)	0.128	2.32	(0.74, 7.31)	0.151
Ever overdosed by accident						
Yes	0.50	(0.19, 1.34)	0.169	2.71	(1.06, 6.91)	0.037
No	1.00	(Ref)		1.00	(Ref)	
Drug-related discrimination by medical community						
Yes	0.25	(0.10, 0.62)	0.003	1.33	(0.55, 3.27)	0.527
No	1.00	(Ref)		1.00	(Ref)	
Ever incarcerated in jail or prison						
Yes	0.31	(0.14, 0.66)	0.003	0.99	(0.40, 2.41)	0.977
No	1.00	(Ref)		1.00	(Ref)	

*Notes* Model adjusted for recruitment source

The log likelihood of the model before stepwise removal is −151.39

The log likelihood of the model after stepwise removal is −157.95

The Nagelkerke R-squared of the model before stepwise removal is 0.478

The Nagelkerke R-squared of the model after stepwise removal is 0.432

The mean variance inflation factor for the model before stepwise removal is 1.33

The mean variance inflation factor for the model after stepwise removal is 1.15

The final model uses multinomial logistic regression and has 16 degrees of freedom

Factors associated with ever being unable to enroll in substance use treatment due to barriers compared to successfully accessing treatment without ever facing barriers are also presented in Table 3. Compared to participants who had successfully enrolled in substance use treatment without ever facing barriers, a history of overdose and a monthly income of $501 - $1500 were associated with an increased adjusted risk of ever unsuccessfully attempting to enroll in substance use treatment. Individuals who reported having ever overdosed by accident had over two and a half times the adjusted risk of having been unsuccessful in an attempt to enroll in substance use treatment compared to successfully enrolling in substance use treatment.

## Discussion

The results of this study demonstrate disparities in access to and utilization of substance use treatment among young adults who use prescription opioids non-medically. Patient-level factors such as race and a history of accidental overdose were correlated with never attempting to enroll in substance use treatment and experiencing barriers while attempting to access these services, respectively.

Although inference is limited by the small sample size, these results nonetheless provide important insights into patient-level barriers to addiction treatment among young adults who report NMPO use, an understudied population regarding access to care. Moreover, our results are consistent with previous studies. A study of 1788 NMPO users ages 12 to 17 found lower levels of perceived need for substance use treatment among black individuals [48], which could be an explanation for the lower levels of enrollment in substance use treatment among non-white participants seen in this study. Alternatively, black individuals may be less likely to ever enroll in treatment because of mistrust of medical professionals due to a history of implicit and explicit racial biases among providers in assessing and undertreating pain and other conditions [49–52].

Previous analysis of provider-level barriers to accessing treatment among street-involved youth and alcohol-dependent men found perceptions of discrimination by the medical community deter treatment seeking and successful enrollment [44, 53]. Though the small sample size limits inference, this conclusion is not supported by the results of our multivariable analysis. However, ever having experienced drug-related discrimination by the medical community was among the strongest correlates of successfully enrolling in substance use treatment compared to never attempting to enroll, suggesting discrimination experiences are, in part, due to an increased level of interaction with the health care system. Although the sample size was not large enough to test interactions between provider- and patient-level factors, it is possible that non-white young adults are more likely to experience drug-related discrimination by the medical community than white young adults. Future research should focus on assessing this disparity during attempts to access substance use treatment, and the ways in which discrimination may be experienced differentially by minority and non-minority young adults. Additionally, medical training should address the impact of drug-related discrimination by the medical community and equip providers with the skills and background necessary to deliver care in an effective, non-discriminatory manner.

Health care system-level factors were also present in the analysis and were found to correlate with access to substance use treatment. Supporting our finding, a previous analysis of barriers to utilizing drug and alcohol treatment among street-involved youth found long waiting lists to be the most common barrier [54]. Additionally, 20 participants (10.0%) in our sample reported that their health insurance did not allow enrollment; the majority (64.1%) of participants who were unsuccessful in enrolling in substance use treatment cited at least one health insurance-related barrier. Nonetheless, insurance status was not found to have an effect on the likelihood of attempting to enroll in substance use treatment in the bivariate analyses. This may be due to high levels of insurance in this sample of young adults (87.5%), which reflects the fact that Rhode Island, as a Medicaid-expansion state, has one of the highest insurance coverage rates in the nation [55]. However, young adults with health insurance may still face financial barriers to accessing substance use treatment due to high deductibles and/or copayments, as well as providers not accepting all types of insurance [36]. Thus, the high rates of health care system-level barriers faced despite a high level of basic health insurance coverage in this sample point towards a need for additional financial assistance programs for young adults who wish to access substance use treatment.

In light of these findings, longitudinal studies are needed to assess the association between changes in insurance type and status over time and access to substance use treatment services. For example, by comparing insurance status and barriers to substance use treatment across time, researchers can assess if individuals experience barriers primarily before gaining insurance, or whether access to care varies as a result of changing insurance provider. This method would be particularly effective in determining the impact of the Affordable Care Act on access to care and treatment-seeking behavior.

With regard to structural factors, our analysis found that past incarceration decreased the likelihood of never enrolling in substance use treatment. This increase in successful enrollments in care is likely due to mandatory or facilitated interaction with the medical system among individuals who were ever incarcerated or detained in a juvenile detention center. Homelessness has previously been found to be independently associated with an inability to enroll in substance use treatment among street-involved youth [43]; this conclusion is supported by the bivariate, but not multivariable, analyses presented here. Homelessness may not be present in the multivariable models due to the inclusion of other structural factors, such as incarceration.

In order to increase access to substance use treatment among young adults who report NMPO use, programs should aim to engage the populations of young adults who are not seeking treatment and those who have unsuccessfully sought treatment. For example, a study of hepatitis C virus treatment in the opioid agonist treatment setting showed the impact of improved health care system organization on access to care. The ETHOS study, which utilized on-site care to improve the treatment pathway and limit the use of unsuccessful referrals, could be extended to treatment of young adults in order to address a system of referrals that is unclear for medical providers treating young adults attempting to begin substance use treatment [36, 56]. Other public health improvements that may result in increased access to substance use treatment include removing age restrictions and/or improving age transitions across a continuum of services, youth-specific housing with integrated addiction treatment services, and peer-led services [44, 57].

### Limitations

This study has a number of limitations that should be noted. First, the study relied on self-reported data, which may be subject to under-reporting or socially desirable reporting biases. To minimize the potential for these biases, sensitive portions of the survey were self-administered using a computer. Second, this study relied on cross-sectional data. This analysis uses lifetime experiences instead of exposures with shorter recall periods (e.g., within the past month) where applicable in an effort to maintain consistency with the recall period for the outcome of interest. Nonetheless, given the cross-sectional nature of our data, precise temporality cannot be ascertained. Therefore, the results of this analysis are purely correlative. We were not able to differentiate between treatment specifically related to opioid use disorder compared to treatment for other drugs, or between an opioid overdose or overdose due to other drug use. Additionally, we did not measure why individuals never attempted to access treatment.

We did not have sufficient sample size to compare (in a multivariable model) individuals who experienced barriers with those who had never experienced barriers. Additionally, the sample size resulted in some small cell sizes, which increased the type II error rate and could have resulted in an inability to detect meaningful differences between groups. Moreover, our use of a stepwise model-building process and multiple tests increased the likelihood of type I error; as such, marginally significant associations should be interpreted with caution. The eligibility criteria used did not include a formal diagnosis of an opioid use disorder, meaning that some participants may not be eligible for certain types of treatment (e.g., opioid agonist therapy). Finally, although diverse methods were used to recruit participants residing throughout the state of Rhode Island, this population was not randomly sampled and does not include participants who are currently involved in formal alcohol or substance use treatment, meaning the sample likely underrepresents individuals who have been successful in accessing substance use treatment. These factors may limit the generalizability of the study outside of young adults who use prescription opioids non-medically and are not currently enrolled in substance use treatment.

## Conclusions

This exploratory study demonstrates significant disparities in access to substance use treatment among young adults who report NMPO use in Rhode Island. Patient-level factors, such as being non-white and past overdose were shown to correlate with never attempting to enroll in substance use treatment and experiencing barriers to substance use treatment utilization, respectively. Public health interventions, including, for example, improved medical training in the management and treatment of substance use disorders among young people and the integration of substance use treatment services within youth-specific shelters and housing programs, are needed to reduce drug-related discrimination in clinical settings and to provide mechanisms that link young adults with treatment.

## Abbreviations

ADD: Attention deficit disorder
ADHD: Attention deficit hyperactivity disorder
GED: General Education Development
MAT: Medication-assisted treatment
MIBR: Mixed internet-based recruitment
NMPO: Non-medical prescription opioid
NSDUH: National Survey on Drug Use and Health
RAPiDS: Rhode Island Young Adult Prescription Drug Study
TARC: Targeted canvassing
